# Group Motivation-Focused Interventions for Patients With Obesity and Binge Eating Disorder

**DOI:** 10.3389/fpsyg.2018.01104

**Published:** 2018-06-29

**Authors:** Giada Pietrabissa

**Affiliations:** ^1^Istituto Auxologico Italiano IRCCS, Psychology Research Laboratory, San Michele Hospital, Milan, Italy; ^2^Department of Psychology, Catholic University of Milan, Milan, Italy

**Keywords:** motivational interviewing, group therapy, weight loss maintenance, obesity, binge eating disorder

Lack of exercise and overeating/unhealthy eating are the main causes of the worldwide escalating global epidemic of overweight and obesity (Pietrabissa et al., 2012)—as defined as a body mass index (BMI) over 30 (Spiegelman and Flier, 2001).

The consequences of excess weight range from serious negative health outcomes, to increased risk of premature death. Obese persons—especially those seeking weight loss treatment (Brennan et al., 2014)—also demonstrate high rates of psychopathology, including mood disorders (i.e., depression, anxiety and low self-esteem) (Luppino et al., 2010) and eating disorders (i.e., binge eating disorder—BED; Succurro et al., 2015).

These result in significantly impaired health-related quality of life (Slagter et al., 2015; Afshin et al., 2017) and loss of productivity (Fontaine and Barofsky, 2001; Robroek et al., 2011). Obesity and its associated problems also have a growing economic impact on the health care system (Hammond and Levine, 2010; Castelnuovo et al., 2016).

The majority of psychological treatments for obese persons with/without BED are cognitive behaviorally based, typically combined with lifestyle interventions (Shaw et al., 2005; Castelnuovo et al., 2017). Still, maintenance of behavioral changes related with weight loss is challenging, and conventional treatments often fail to prevent relapses (Wu et al., 2009; Castelnuovo et al., 2011).

The most common reasons for withdrawal are low motivational status and self-efficacy (i.e., belief in one's ability to perform the behavior), as well as unrealistic weight-loss expectations (Teixeira et al., 2012b). Notably, obese binge eaters are more likely to drop out of weight-loss treatments, and to regain lost weight faster than non-binge eaters (Marcus et al., 1988; Rieger et al., 2005), thus reporting worse psychological functioning and lower confidence in their abilities than obese individuals without BED (Cargill et al., 1999; Clark et al., 2000).

In fact, existing weight loss programs place tremendous emphasis on behavioral skills refinement. However, people usually report that they know what to do to control their weight, but cannot help themselves to continue implementing healthy habits (Pietrabissa et al., 2015).

This has prompted the adoption of various strategies to increase maintenance of behavioral changes associated with weight loss (Leavey et al., 2011).

Research has shown motivational interviewing (MI) to be effective in promoting behavioral change across a range of health arenas (Dunn et al., 2001; Burke et al., 2003; Heckman et al., 2010; Yakovenko et al., 2015; Alperstein and Sharpe, 2016; Dillard et al., 2017), including weight/body mass index (BMI) reduction (West et al., 2007; Armstrong et al., 2011; DiLillo and West, 2011; Barnes and Ivezaj, 2015; Borrello et al., 2015; Mirkarimi et al., 2017), increased physical activity (Brodie and Inoue, 2005; Letourneau and Goodman, 2014; O'Halloran et al., 2014; Bean et al., 2015; Soderlund, 2018) and binge episodes decrease (Cassin et al., 2008; Vella-Zarb et al., 2015).

MI is a collaborative, client-centered counseling approach for enhancing intrinsic motivation to change by eliciting *change talk* (the individuals' own reasons for change—key feature of MI) and exploring *ambivalence* about behavioral change while trying to examine discrepancies between the individuals' current behavior and their personal goals and values (Rollnick et al., 1999). MI has been linked to constructs from several social-psychological models of health behavior. It is consistent with *self-determination theory* (Deci and Ryan, 2012; Patrick and Williams, 2012; Teixeira et al., 2012a; Vansteenkiste et al., 2012; Phillips and Guarnaccia, 2017), which suggests that successful long-term weight maintenance is expected when levels of autonomous motivation and confidence for behavior change are high (Deci and Ryan, 1985; Miller and Rollnick, 2012).

According to the *social cognitive theory*, MI strategies also serve to enhance individuals' self-efficacy, which is not only an essential element in motivation, but also a good predictor of treatment outcome (Bandura, 1977). Finally, MI is linked to increased readiness to change from the *transtheoretical model* (Wilson and Schlam, 2004; Dray and Wade, 2012; Braillon and Taiebi, 2016). Within this framework, behavioral change is conceptualized as a five-stage process (1. Precontemplation: subjects do not consider changing their unhealthy lifestyle; 2. Contemplation: subjects are thinking—but they are still ambivalent—about a possible lifestyle change; 3. Determination: subjects are planning/getting ready to make the change; 4. Action: subjects are doing something to change their unhealthy lifestyle; 5. Maintenance: subjects are consolidating the change over time) related to the individuals' self-confidence in their abilities and intrinsic motivation to change. Relapses are considered an integral part of movement toward sustained change (Prochaska and Velicer, 1997), since self-efficacy improves as people experience failure and learn to succeed by overcoming obstacles.

In evidence-based multi-componential weight loss interventions individual therapy (IT) and group therapy (GT) are often combined to produce optimal outcomes. They are not at odds with each other, but the issues addressed in individual sessions complement those of GT, and vice versa.

Groups provide members with a meaningful social support system alternative perspectives, and interpersonal feedbacks in a cost-effective way (Velasquez et al., 2006), and allow the health care providers (HCPs) to address common concerns and to build off strengths and experiences of multiple clients simultaneously (Forsyth, 2011; Tasca et al., 2012; Grenon et al., 2017). It has been reported that perception of alliance between obese group members is more likely to produce favorable outcomes (Gullo et al., 2014) than the existence of a positive relationship between the members and the HCPs (Kivlighan et al., 2017). Treatment options (IT or GT) could be responsible for different outcomes in health care, but research evidence of those compared for weight problems is still contradictory and incomplete (Hakala et al., 1993; Renjilian et al., 2001; Minniti et al., 2007) and no study has yet specifically confronted the effectiveness of MI in enhancing lifestyles modification when delivered in group rather than in IT.

This is partly due to the fact that, despite the popularity of MI in individual weight loss counseling and the appeal of adapting MI for GT with patients suffering from obesity and BED, information on how to apply MI in groups are still scant.

In MI two active ingredients are present: a relational and a technical component. The spirit or mindset of MI is concerned with the relational aspect and encompasses a *collaborative* group environment focused on understanding the unique perspectives and needs of each member. The use of evocative questions can help the HCPs engage group members and evoke change from within rather than teach skills or provide psychoeducation. Information are given only asking permission (Miller and Rollnick, 2002), and the elicit (what they already know on the topic)-provide (reactions)-elicit framework is particulate useful to check members' understanding and to promote active learning.

Each group member is encouraged to share expectations, concerns and change success stories—thus to bolster self-efficacy (Miller and Rollnick, 2002).

Still, quite often there are thing that are more important to people than making health behavior change. Exploration of the unique values and of the pros and cons of both changing and not making a change (*decisional balance)* are powerful tools by attending to change talk responses and resolving ambivalence to change (Janis and Mann, 1977; Miller and Rollnick, 2002).

*Reflective listening* and *summaries* of the group members' statements and feelings back to them are also likely to facilitate and shift the discussion to fruitful topics—including *affirmation* of personal choices and responsibility for alternative behaviors, as well as underpinning of a change plan consisting of small, manageable goals (Wagner and Ingersoll, 2013).

The ground rules of the group should be made explicit at the beginning of each session, and hostile or dominating speech discouraged in favor of deliberate commitment to pursue the welfare and best interest of the others (*compassion*) in an empathic atmosphere of *acceptance* and respect for individual differences. Group members are, in fact, often diverse in terms of their target behaviors, stages of change, and reasons for change. Still, group's heterogeneity is believed to be a curative factor as far as common important themes emerge (Yalom, 1995). Asking group members to comment on the positive attributes and strengths of others (*eliciting strengths*) may further help reducing discord (Wagner and Ingersoll, 2013).

Notably, despite overweight and obese individuals usually describe themselves as highly motivated in making the change, confidence in their ability to do so and to maintain the achieved results is often lacking. Persons may feel demoralized because they attempted to change many times, but relapses of weight-management behaviors following intentional weight loss are commonly observed.

The *readiness ruler* (RR) is a helpful tool to be used in order to assess patients' confidence and readiness to change during treatment, and to promote the identification and discussion of perceived barriers to change (St-Hilaire et al., 2017).

It is clear that HCPs employing MI in a group setting should be highly skilled and experienced both in MI and in GT, as successful adaptation requires attention to complex individual change processes as well as group dynamics, and the interplay between the two.

Despite the promising findings for the efficacy of MI in promoting lifestyle changes (Rubak et al., 2005; Zomahoun et al., 2017), results for maintaining weight loss over a long period of time are still unclear due to the absence of reporting of the effects of MI interventions beyond 6-months (Teixeira et al., 2012b).

In a world that is increasingly relying on technology (e.g., cell phones, email, Internet) to communicate, telemedicine-based weight loss program may enable individuals to access to more information and to increase their ability to self-monitor with greater ease compared with traditional methods (Castelnuovo et al., 2015).

While on-line MI face-to-face interventions are becoming popular due to their preliminary promise in promoting positive health outcomes in the outpatient settings (Friederichs et al., 2015; Karnes et al., 2015), additional investigations are needed to test the goodness of web-based MI group sessions. In fact, both online-IT and online-GT are convenient in their capacity to involve hard to reach people (Sorgente et al., 2017), and GT may offer several benefits over individual sessions, such as anonymity, reduced embarrassment and stigma, and greater members' openness and self-disclosure (Osilla et al., 2012). To date, only a pilot study has been carried out to test online group MI among 20 adults with obesity (Webber et al., 2008), providing preliminary evidence on its usefulness for weight reduction. Still, the investigation was uncontrolled, with a small sample size.

Moreover, research often do not shed light on the effectiveness of MI as a stand-alone treatment, but in conjunction with other psychosocial interventions (Cassin et al., 2008; DiMarco et al., 2009; Karlsen et al., 2013; Vella-Zarb et al., 2015; Moss et al., 2017; Pietrabissa et al., 2017), therefore limiting the investigation of the specific impact of MI on weight loss maintenance determinants. A very little evidence also exists on MI treatment fidelity, including information as to how adherence to the intervention is assured.

In summary, MI interventions are likely to promote sustained behavior change, yet further research is needed to study MI in the group setting and to deepen the investigation of the mechanisms selected to influence long-term health outcomes. Empirical evidence supports theoretical knowledge that high level of self-efficacy responds confidently to behavioral barriers, beside improving the individuals' motivation to persevere in the pursuit of goals in spite of the setbacks that periodically test their willpower (Berman, 2006; Bonsaksen et al., 2012).

Theory-driven, evidence-based group strategies aimed at cost-effectively improving individuals' self-efficacy and self-management capacity may, therefore, have a significant impact in improving the management of people with weight problems.

## Author contributions

The author confirms being the sole contributor of this work and approved it for publication.

### Conflict of interest statement

The author declares that the research was conducted in the absence of any commercial or financial relationships that could be construed as a potential conflict of interest.
